# *Panax notoginseng*-Derived Carbon Quantum Dots as a Novel Administration Strategy for Cerebral Ischemia–Reperfusion Injury Therapy

**DOI:** 10.34133/bmr.0440

**Published:** 2026-10-29

**Authors:** Wen-Qiang Cui, Sheng Wei, Hai-Tao Li, Hong-Wei Zhi, Jin-Ping Zhang, Yu Wang, Zhen-Ling Liu, Feng-Xian Hu, Xiang-Qing Xu, Hong-Yun Wu

**Affiliations:** ^1^ Department of Neurology, Affiliated Hospital of Shandong University of Traditional Chinese Medicine, Jinan 250014, PR China.; ^2^ First College of Clinical Medicine, Shandong University of Traditional Chinese Medicine, Jinan 250355, PR China.; ^3^ Xiyuan Hospital of China Academy of Chinese Medical Sciences, Beijing 100091, PR China.; ^4^ Institute for Chinese Medicine and Brain Science, Shandong University of Traditional Chinese Medicine, Jinan 250355, PR China.; ^5^ Key Laboratory of Traditional Chinese Medicine Classical Theory, Ministry of Education/Shandong Key Laboratory of Innovation and Application Research in Basic Theory of Traditional Chinese Medicine/Shandong Provincial Engineering Research Center for the Prevention and Treatment of Major Brain Diseases with Traditional Chinese Medicine, Shandong University of Traditional Chinese Medicine, Jinan 250355, PR China.

## Abstract

The present study aimed to synthesize *Panax notoginseng*-derived carbon quantum dots (PN-CDs) and explore their role in cerebral ischemia–reperfusion injury (CIRI). PN-CDs were synthesized by the hydrothermal method, and characterization and biocompatibility were assessed. Next, CIRI models were established using SH-SY5Y cells, primary neurons were treated with oxygen-glucose deprivation/reoxygenation (OGD/R), and rats were subjected to middle cerebral artery occlusion/reperfusion (MCAO/R). After PN-CDs treatment, inflammation, apoptosis, and oxidative stress were detected in neuronal cells and rats using enzyme-linked immunosorbent assay, quantitative reverse transcription polymerase chain reaction, flow cytometry, Western blot, and 2′,7′-dichlorodihydrofluorescein diacetate probe. Meanwhile, 2,3,5-triphenyltetrazolium chloride, terminal deoxynucleotidyl transferase dUTP nick end labeling, Nissl, and hematoxylin and eosin staining were employed to measure the pathological damage of rats. Additionally, CIRI rats were administered with PN-CDs, *Panax notoginseng* saponins (PNS), *Panax notoginseng* (PN), or edaravone to investigate whether PN-CDs had the best effect. The findings revealed that PN-CDs exhibited favorable biocompatibility and crossed the blood–brain barrier. In vitro, PN-CD treatment inhibited oxidative stress, apoptosis, and inflammation, as evidenced by the decrease of interleukin-1β (IL-1β), IL-6, Bax, C-caspase-3, tumor necrosis factor-α, malondialdehyde, and reactive oxygen species and the increase of superoxide dismutase, Bcl-2, and glutathione. In CIRI rats, PN-CDs improved pathological damage and suppressed inflammation, apoptosis, and oxidative stress. The therapeutic effect of PN-CDs on CIRI was distinctly better than that of PNS and PN, and comparable to that of the first-line drug edaravone. In short, PN-CDs, a novel and efficient administration strategy, alleviates CIRI by inhibiting apoptosis, oxidative stress, and inflammation.

## Introduction

Stroke, a prevalent cerebrovascular disorder with substantial morbidity and mortality, arises from cerebral vessel occlusion or rupture, which ultimately leads to focal or global brain tissue damage. Hemorrhagic stroke and ischemic stroke are recognized subtypes of strokes. In particular, ischemic stroke constitutes approximately 80% of total strokes and has become the main subtype. Ischemic stroke not only impairs the patient’s language, action, sensory, and cognitive functions, but also imposes a heavy financial burden on families. While considerable progress has been made in the diagnosis and management of ischemic stroke, the therapeutic efficacy is still inadequate. So far, rtPA has been approved for treating ischemic stroke, but it has a strict time window of 4.5 h after stroke [1]. It is worth noting that rt-PA often induces cerebral ischemia–reperfusion injury (CIRI) in the process of dissolving thrombus and restoring blood flow [2]. At present, a variety of neuroprotective agents are being studied in preclinical research. Although numerous promising neuroprotective agents have exhibited robust therapeutic effects in preclinical animal models, they fail to deliver satisfactory functional outcomes in clinical settings [3,4]. Consequently, the vast majority of these drugs have not been translated into routine clinical use. Their major therapeutic limitations include inadequate blood–brain barrier (BBB) penetration, low bioavailability, and pronounced systemic adverse effects [3,5]. Additionally, the pathogenesis of CIRI is intricate, and may involve inflammation, apoptosis, oxidative stress, and ion imbalance. Hence, it is urgently necessary to clarify the pathological mechanisms and develop effective treatment strategies for CIRI.

*Panax notoginseng* (PN), a highly prized Chinese herbal medicine predominantly planted in Yunnan and Guangxi of China, exhibits multiple pharmacological properties, such as hemostasis, anti-inflammation, vasodilation, and enhancing blood flow to eliminate blood stasis, and has been extensively utilized to treat cerebrovascular and cardiovascular diseases. Saponins, polysaccharides, and flavonoids are the key active components of PN. Recently, the role of *Panax notoginseng* saponins (PNS) in CIRI has attracted extensive attention from researchers. Wang et al. [6] observed that PNS suppressed ferroptosis and inflammation in CIRI. In CIRI rats, PNS treatment promoted mitophagy, inhibited the activation of inflammasome NLRP3, and alleviated brain tissue damage via the PINK1/Parkin pathway [7]. PNS also inhibited oxidative stress, enhanced neurological function, and reduced infarct volume and histopathological damage in CIRI [8]. However, the bioavailability of PN and PNS is low due to large molecular weight and poor water solubility.

With the continuous development of science and technology, nanotechnology has brought a new dawn to the medical field. Composed of carbon cores and surface passivation layers, carbon quantum dots (CQDs) are a category of zero-dimensional carbon nanomaterials with a diameter of less than 10 nm and inherent fluorescent properties. In the field of catalysis, CQDs have been verified to exhibit high catalytic efficiency and mimic enzymatic activities. The catalytic performance of CQDs is mainly attributed to their abundant surface oxygen-containing functional groups and large sp^2^ carbon domains [9]. Additionally, the rich oxygenated moieties on CQDs surfaces also confer superior water solubility to these nanomaterials. Recently, CQDs have been extensively employed in disease diagnosis and treatment and biological imaging due to their easy preparation, good biocompatibility, small size, and low toxicity [10]. In particular, the synthesis of CQDs from biomass waste has emerged as a green and sustainable strategy. For instance, biomass-derived CQDs have been successfully applied in biological imaging [11], heavy metal ion detection [12], and photocatalysis fields [13], indicating that they possess great multifunctional potential. Benefiting from green synthesis routes, low production cost, and potent reactive oxygen species (ROS) scavenging capacity, natural medicine-derived CQDs have garnered tremendous attention among clinical researchers [14]. This strategy also holds great promise for addressing the poor solubility and low bioavailability inherent to natural medicinal compounds. Zhang et al. [15] synthesized CQDs derived from Crinis Carbonisatus and found that it has inhibitory effects on the levels of tumor necrosis factor-α (TNF-α) and interleukin-6 (IL-6) and that it improved neurological function and alleviated pathological damage in CIRI rats. CQDs isolated and identified from *Gardenia jasminoides* inhibited inflammation and oxidative stress, reduced infarct volume, and alleviated CIRI disease progression [16]. Existing studies suggest that natural drug-derived CQDs have unique advantages in the treatment of CIRI. So far, scholars have developed numerous CQD synthesis strategies (e.g., hydrothermal method, solvothermal method, electrochemical oxidation method, and laser ablation method) [17,18]. Interestingly, the size, surface chemical, and optical properties of CQDs were precisely controlled by adjusting synthesis conditions, thereby endowing them with diverse functionalities, which is crucial for their application in the biomedical field [19,20].

Herein, we adopted PN as a natural drug-derived precursor to fabricate CQDs, given its favorable pharmacological properties and superior biocompatibility. Next, CQDs derived from *Panax notoginseng* (PN-CDs) were added to CIRI rat and cell models, and then evaluated inflammation, apoptosis, oxidative stress, and pathological damage to explore its effect. These results may bring a novel dawn for the clinical therapy of CIRI.

## Materials and Methods

### PN-CDs synthesis

In order to synthesize PN-CDs, the hydrothermal method was performed. Firstly, deionized water (10 ml) was mixed with PN powder (0.2 g), followed by sonication for 10 min to achieve complete dissolution. Subsequently, the above solution was transferred to a stainless-steel autoclave with a polytetrafluoroethylene liner and reacted at 200 °C for 12 h. Notably, we systematically evaluated the quantum yield (QY), particle size distribution, and dispersion stability of PN-CDs at different hydrothermal temperatures (160, 180, 200, and 220 °C) and reaction times (6, 12, 18, and 24 h) to identify the optimal synthesis conditions (200 °C, 12 h) [11,12,21,22]. After the solution was cooled to 25 °C, macromolecular impurities were removed by high-speed centrifugation and ultrafiltration. To further separate PN-CDs from low-molecular-weight impurities, the solution was dialyzed against ultrapure water for 72 h using a 1,000-Da dialysis bag, with water changes every 12 h. Finally, PN-CDs was obtained using vacuum freeze-drying. To ensure the repeatability between batches, we evaluated the morphology, particle size, surface charge, fluorescence properties, and stability of PN-CDs synthesized in 3 separate random batches.

### PN-CDs characterization

The particle size and morphology of PN-CDs were detected by a transmission electron microscope (TEM, JEM-F200, JEOL, Japan) and an atomic force microscope (AFM, Dimension Icon, Bruker, Germany). Ultraviolet–visible spectroscopy (UV–Vis, UV-3600i Plus, SHIMADZU, Japan) and photoluminescence (PL, F-4600, Hitachi, Japan) were employed to evaluate the optical behavior of PN-CDs. The functional groups and chemical elements of PN-CDs were analyzed using Fourier transform infrared spectroscopy (FTIR, IRTracer-100, SHIMADZU, Japan) and x-ray photoelectron spectroscopy (XPS, K-Alpha, Thermo Fisher Scientific, USA). X-ray diffraction (XRD, D8 ADVANCE, Bruker, Germany) and Raman (DXR2xi, Thermo Fisher Scientific, USA) were employed to assess the crystal structure and order of PN-CDs. The surface charge and stability of PN-CDs were detected by Zeta potential (Zetasizer Pro, Malvern Panalytical, USA). Under a steady-state/transient fluorescence spectrometer (FLS 1000, Edinburgh, Britain), the QY of PN-CDs at 439-nm excitation wavelength was determined by integrating sphere (inner surface coated with polytetrafluoroethylene) absolute method.

### Animals

Sprague–Dawley (SD) rats (male, 8 weeks old) were supplied by Jinan Pengyue Experimental Animal Breeding Co., Ltd. Rats were housed in a standard environment for 7 d, during which water and food were provided ad libitum.

### CIRI rat model construction and drug treatment

The SD rats were allocated into 10 groups: control, PN-CDs, sham, CIRI, CIRI + L-PN-CDs, CIRI + M-PN-CDs, CIRI + H-PN-CDs, CIRI + PNS, CIRI + PN, and CIRI + Edaravone. The MCAO/R model of rats in the CIRI group was established using the modified Zea Longa method as reported in an earlier study [23,24]. Briefly, rats were anesthetized and fixed on the operating table. At the midline of neck, a 1-cm incision was created using surgical scissors. After making an incision on the right common carotid artery, a nylon thread was inserted and pushed along the internal carotid artery to the intracranial until it encountered a certain resistance. Blood flow was restored by withdrawal of the suture after 2 h of ischemia. Additionally, penicillin was injected into the rats for 2 consecutive days. Sham-operated rats underwent identical surgical procedures without arterial occlusion. After MCAO/R modeling for 24 h, rats in CIRI + L-PN-CDs, CIRI + M-PN-CDs, CIRI + H-PN-CDs, CIRI + PNS, and CIRI + PN groups were orally administered with PN-CDs (100, 200, and 300 mg/kg/d), PNS (120 mg/kg/d), and PN (300 mg/kg/d) for 7 consecutive days, respectively. Rats in the CIRI + Edaravone group were intraperitoneally injected with edaravone (HY-B0099, MedChemExpress, USA, 6 mg/kg/d) for 7 consecutive days. Rats in sham and CIRI groups were orally administered with an equal volume of phosphate-buffered saline (PBS) for 7 consecutive days. At 8 d after MCAO/R surgery, neurological function was evaluated in rats, which were then euthanized for brain tissue collection and further analyses. Notably, rats in PN-CDs and control groups were orally administered with PN-CDs (300 mg/kg/d) or the same amount of PBS, without MCAO/R surgery. After 7 d of continuous administration, rats were euthanized.

### Cell culture

SH-SY5Y cells supplied by the Cell Bank of Chinese Academy of Sciences (Shanghai, China) were cultured in DMEM/F12 medium (11320033, Gibco, USA) with 10% fetal bovine serum (FBS, A5670701, Gibco, USA).

Primary neurons were extracted from embryonic day 18 (E18) of SD rats [25,26]. Briefly, the cerebral cortex tissues were separated, placed in a precooled Hanks’ balanced salt solution (E607005, Sangon Biotech, China) and cut into pieces. After digesting with papain (A425953, Sangon Biotech, China) supplemented with DNase I (90083, Thermo Fisher Scientific, USA), samples were added with DMEM/F12 medium containing 1% penicillin/streptomycin and 10% FBS to terminate the reaction. Subsequently, a 70-μm cell strainer was filtered to filter cell suspension. DMEM/F12 medium containing 1% penicillin/streptomycin and 10% FBS was prepared for resuspending the cells. After the cells were cultured for 4 h, the above medium was substituted with Neurobasl-A medium (10888022, Thermo Fisher Scientific, USA) containing 1% Gluta-MAX (35050061, Thermo Fisher Scientific, USA) and 2% B-27 (17504044, Thermo Fisher Scientific, USA), transferred to poly-d-lysine-coated 6-well plates (152035, Thermo Fisher Scientific, USA), and then cultured for 7 d to obtain primary neurons.

### OGD/R induction and treatment

Under hypoxic conditions (94% N₂, 5% CO₂, and 1% O₂), cells were maintained in serum-free low-glucose medium at 37 °C for 2 h. Then, complete medium was applied to replace the original culture fluid. To generate the in vitro oxygen-glucose deprivation/regain (OGD/R) cell model, cells were maintained for 24 h under normoxic conditions (74% N₂, 5% CO₂, and 21% O₂).

OGD/R-induced primary neurons and SH-SY5Y were treated with 40, 160, and 640 μg/ml PN-CDs for 24 h and collected for subsequent experiments. To explore the biocompatibility of PN-CDs, SH-SY5Y and primary neurons were treated with 0, 10, 20, 40, 80, 160, 320, 640, and 1,000 μg/ml PN-CDs.

### CCK-8 assay

SH-SY5Y and primary neurons seeded in 96-well plates were exposed to PN-CDs, and then treated with CCK-8 reagent (A311-01, Vazyme, China) of 10 μl in a 37 °C incubator. Two hours later, sample absorbance was measured at 450 nm.

### Calcein-AM/PI double staining

Calcein-AM/PI Double Stain Kit (40747ES76, Yeasen, China) was utilized to measure cell viability. As described in the kit instructions, working solution was prepared by adding 5 ml of 1 × assay buffer to PI (1.5 mM, 15 μl) and Calcein-AM (2 mM, 5 μl). Next, primary neurons and SH-SY5Y were incubated with PN-CDs and treated with the above working solution for 20 min. The living (green) and dead (red) cells were recorded.

### Hemolysis test

The whole blood of SD rats was collected and centrifuged to isolate red blood cells (RBCs). To prepare 2% RBC solution, the obtained RBCs were mixed with normal saline. Subsequently, deionized water (positive control), PN-CDs, and normal saline (negative control) were incubated with 2% RBC solution for 4 h, respectively.

### Establishment of BBB model in vitro

Primary brain microvascular endothelial cells (BMECs) and astrocytes of SD rats were isolated to establish a BBB model in vitro [27,28]. Before the experiment, Transwells (Beijing Noble Ryder Technology Co., Ltd, China) were pretreated with poly-l-lysine (A600751, Sangon Biotech, China) and collagen I (A1048301, Thermo Fisher Scientific, USA) for 3 h, and then dried in an ultra-clean bench. Astrocytes were seeded in the lower layer of Transwell and incubated for 4 h to adhere to the surface of Transwell. Next, BMECs were seeded on the upper layer of Transwell. A Millicell-ERS 3.0 (Millipore, USA) was used to measure the trans-epithelial electrical resistance (TEER) of the RBMEC monolayer. When TEER reached 200 Ω·cm^2^, the subsequent experiments were performed.

### Evaluation of BBB permeability of PN-CDs

Unlike PN-CDs, PN exhibits nonfluorescent properties. To explore the BBB permeability of PN-CDs and PN, Cy5.5 (HY-D0924, MedChemExpress, USA) was utilized for labeling PN-CDs and PN via electrostatic adsorption. Briefly, PN-CDs or PN was adequately mixed with Cy5.5 dissolved in dimethyl sulfoxide, and then incubated in darkness with shaking for 2 h. Next, different concentrations of PN-CDs or PN (40, 160 and 640 μg/ml) were added to the upper layer of the BBB model. At 30, 60, and 120 min, the solution containing PN-CDs or PN in the lower layer was collected into a 96-well plate and the fluorescence intensity was detected through a fluorescence microscope (Olympus, Japan). Meanwhile, PBS was filled into the lower layer of Transwell to replace the collected solution.

### Blood biochemistry assay

The whole blood of rats in control and PN-CDs groups was collected and centrifuged to isolate serum. The concentrations of total bilirubin (TBIL), alanine aminotransferase (ALT), alkaline phosphatase (ALP), uric acid (UA), direct bilirubin (DBIL), aspartate aminotransferase (AST), blood urea nitrogen (BUN), and lactate dehydrogenase 1 (LDH1) in serum were detected by the TBIL Colorimetric Assay Kit (K095, Fine Test, Wuhan, China), the DBIL Colorimetric Assay Kit (abs580260, Absin, Shanghai, China), the ALP Assay Kit (A059-2-2), the Urea Assay Kit (C013-2-1), the AST Assay Kit (C010-2-1), the ALT Assay Kit (C009-2-1), the UA Test Kit (C012-2-1) (Nanjing Jiancheng Bioengineering Institute, China), and the LDH1 Assay Kit (ab197000, Abcam, USA).

### Hematoxylin and eosin staining

Rat tissues fixed in 4% paraformaldehyde were washed several times with tap water. Then, tissues were embedded in paraffin and cut into slices for subsequent research. Hematoxylin and eosin (H&E) Staining Kit (EE0012, SparkJade, China) was applied to assess the pathological damage of tissues. As described in the kit instructions, tissues were incubated with hematoxylin (5 min) and then stained with eosin (1 min). After dehydration, transparency, and seal sheet, tissues were observed.

### ELISA analysis

Neuronal cells and brain tissues were lysed and centrifuged to collect cell supernatants and tissue homogenates. Then, IL-1β, TNF-α, and IL-6 levels were detected with the IL-1β enzyme-linked immunosorbent assay (ELISA) Kit (Human: ml028592; Rat: ml037361), the TNF-α ELISA Kit (Human: ml106471; Rat: ml002859), and the IL-6 ELISA Kit (Human: ml058097; Rat: ml064292), respectively. All kits were supplied by Shanghai Enzyme-linked Biotechnology Co., Ltd. (China).

### qRT-PCR

Total RNA was isolated using TRIzol reagent supplied by Thermo Fisher Scientific (catalog number 15596018CN, USA). Next, the cDNA Reverse Transcription Kit (4374966, USA) purchased from Thermo Fisher Scientific was utilized to convert total RNA into complementary DNA through reverse transcription. PCR was carried out using a quantitative reverse transcription polymerase chain reaction (qRT-PCR) kit (11732020, Thermo Fisher Scientific, USA). As shown in Table 1, the primer sequences are listed. The level of genes was analyzed using the 2^−ΔΔCt^ method [24].

**Table 1.** Forward and reverse primer sequences for qRT-PCR

Gene	Forward primer (5′-3′)	Reverse primer (5′-3′)
Human-TNF-α	GCCCATGTTGTAGCAAACCC	TGAGGTACAGGCCCTCTGAT
Human-IL-1β	AACCTCTTCGAGGCACAAGG	GTCCTGGAAGGAGCACTTCAT
Human-IL-6	TCCTTCTCCACAATACCCCCA	GGTTGTTTTCTGCCAGTGCC
Human-GAPDH	AATGGGCAGCCGTTAGGAAA	GCCCAATACGACCAAATCAGAG
Rat-TNF-α	GATCGGTCCCAACAAGGAGG	CTTGGTGGTTTGCTACGACG
Rat-IL-1β	CAGCTTTCGACAGTGAGGAGA	TTGTCGAGATGCTGCTGTGA
Rat-IL-6	CCAGTTGCCTTCTTGGGACT	TGCCATTGCACAACTCTTTTC
Rat-GAPDH	AAGGTCGGTGTGAACGGATTT	CAGCTTCCCATTCTCAGCCT

### Western blot

After extracting total proteins via radioimmunoprecipitation assay solution, samples were separated by electrophoresis and transferred. Then, the membrane was blocked, incubated with primary antibodies against Bax (AF0120), IL-6 (DF6087), Bcl-2 (AF6139), TNF-α (AF7014), C-caspase-3 (AF7022), IL-1β (AF5103), and GAPDH (AF7021) overnight at 4 °C, and then incubated with secondary antibody (S0001) at 37 °C for 1 h. Proteintech (China) provided all antibodies. Finally, the ECL Luminescence Kit (ED0015, China) purchased from SparkJade was employed to visualize protein bands.

### DCFH-DA assay

DCFH-DA (2′,7′-dichlorodihydrofluorescein diacetate) probe (S1105S, Beyotime Biotechnology, China) was utilized to evaluate the level of ROS in cells. After the specific treatment, SH-SY5Y and primary neurons were treated with 10 μM DCFH-DA probe and washed. Images were documented. All operations were carried out in the dark.

### Determination of SOD, GSH, and MDA

After the specific treatment, neuronal cells and brain tissues were collected, lysed, and centrifuged to obtain cell supernatants and tissue homogenates. The levels of glutathione (GSH), superoxide dismutase (SOD), and malondialdehyde (MDA) in tissues and cells were measured using the GSH Assay Kit (EEA020), the SOD Assay Kit (EIASODC), and the MDA Assay Kit (EEA015), respectively. Thermo Fisher Scientific (USA) provided all kits. The absorbance of samples was determined. The wavelengths of MDA, SOD, and GSH were 532, 450, and 405 nm, respectively.

### Flow cytometry analysis

The Annexin V-FITC/PI Apoptosis Detection Kit (40302ES50, Yeasen, China) was employed to evaluate neuronal cell apoptosis. After the specific treatment, SH-SY5Y and primary neurons were added with Annexin V-FITC (5 μl) and PI staining solution (10 μl), mixed, and reacted for 15 min. This process was carried out in darkness.

### Neurological function assessment

The Zea Longa method was adopted to assess the neurobehavioral function of rats [29]. The scoring criteria were as follows: 4, loss of consciousness and spontaneous movement; 3, keep tilting to the right; 2, keep circling to the right; 1, failure to fully extend the right front paw; 0, no neurological dysfunction.

### TTC staining

The brain tissue of rats was collected, washed, frozen, and cut into sections (2 mm). After incubating with 2% 2,3,5-triphenyltetrazolium chloride (TTC) (60508ES25, Yeasen, China), the tissue sections were washed and fixed in 4% PFA. Normal tissues were marked as red, while infarcted tissues were marked as white. The percentage of infarcted volume to total volume was counted.

### Nissl staining

Rehydrated and deparaffinized brain tissue sections were labeled with Nissl solution (C0117, Beyotime Biotechnology, China). After treatment for 30 min, the sections were washed, dehydrated, transparent, and then sealed with neutral gum. Images were observed and recorded.

### TUNEL staining

The apoptosis of rat brain tissues was detected by terminal deoxynucleotidyl transferase dUTP nick end labeling (TUNEL) Kit (C1089, Beyotime Biotechnology, China). Sections were maintained in proteinase K (20 μg/ml) without DNase and washed several times with PBS. Next, TUNEL working solution was prepared and treated with the sections for 1 h. Images were obtained and recorded.

### Statistical analysis

GraphPad Prism 6 software was employed to conduct all statistical analyses. To evaluate the difference, one-way analysis of variance and unpaired *t* test were implemented. The standard of significant difference was that *P* < 0.05. Data were presented as mean ± SD.

## Results

### Synthesis and characterization of PN-CDs

As revealed in Fig. 1A, PN-CDs were synthesized from PN by the hydrothermal method. Then, we evaluated the characterization of PN-CDs, including morphology, particle size, chemical element composition, functional groups, optical properties, crystal structure, and stability. TEM results revealed that PN-CDs were uniformly dispersed spherical nanoparticles with an average diameter of 2.32 ± 0.47 nm and a lattice fringe spacing of 0.21 nm, which represented the (100) crystal plane of carbon (Fig. 1B). Consistently, AFM images showed that PN-CDs had good dispersibility and a thickness of 2.5 nm (Fig. 1C). The XRD patterns of PN-CDs exhibited broad peaks at 2*θ* = 42° and 2*θ* = 28°, which coincided with the (100) and (002) planes of carbon, respectively (Fig. 1D). Peak broadening and decreased intensity reflected partial graphitization with marked disorder, and the 42° faint peak provided further evidence of abundant amorphous carbon in the structure. The results confirm that the synthesized CDs exhibit a heterogeneous, multilayered structure containing discrete crystalline regions, which is consistent with disordered carbon [30,31]. In Raman, the characteristic peaks at 1,350 and 1,580 cm^−1^ correspond to D and G bands, respectively. The intensity ratio of D band to G band (ID/IG) of PN-CDs was 0.82, indicating that it exhibited a relatively high graphitization degree and abundant surface defect (Fig. 1E). The well-developed π–π planar structure accelerates electron transfer, and the residual defect sites serve as active centers, which endows PN-CDs with favorable catalytic performance [32,33]. Moreover, the UV–Vis results of PN-CDs indicated that absorption peaks appeared at 270 and 330 nm, corresponding to the π–π* transition of the conjugated C=C bond in the carbon core and the n–π* transition of surface functional group [34] (Fig. 1F). Additionally, PN-CDs had certain stability and its Zeta potential was −13.39 ± 0.34 (Fig. 1G). XPS was used to detect elemental composition of PN-CDs. As displayed in Fig. 1H, PN-CDs were mainly composed of carbon, oxygen, and nitrogen. High-resolution spectra identified the chemical states with C1s displayed components for C=C (284.8 eV, 71.7%), C–N (286.4 eV, 20.6%), and C=O (288.2 eV, 7.7%); O1s contributions for C=O (531.2 eV, 31.9%) and C–O (532.5 eV, 68.1%); and N1s elements for pyridinic N (399.1 eV, 24.1%), pyrrolic N (399.9 eV, 45.1%), and graphitic N (401.1 eV, 30.8%). FTIR analysis was subsequently employed to characterize the surface functional groups of PN-CDs. The results showed that characteristic peaks appeared in 3,428, 2,940, 1,657, 1,405 and 1,106 cm^−1^, corresponding to O–H, C–H, C=O, C–N, and C–O, respectively [35] (Fig. 1I). The C–N peak at 1,405 cm^−1^ indicates the successful incorporation of nitrogen into the carbon skeleton. This nitrogen doping most likely originates from the nitrogen-rich endogenous components of PN, such as proteins, amino acids, and alkaloids, which serve as natural nitrogen sources during the hydrothermal synthesis [36]. PN-CDs exhibited an emission wavelength of 521 nm and an excitation wavelength of 439 nm (Fig. 1J). The green fluorescence emission endows PN-CDs with great potential in visual detection and optical labeling. QY is a key parameter for evaluating the performance of luminescent materials. In this study, the absolute QY of PN-CDs was 7.83%, suggesting favorable fluorescence conversion efficiency.

**Fig. 1.**
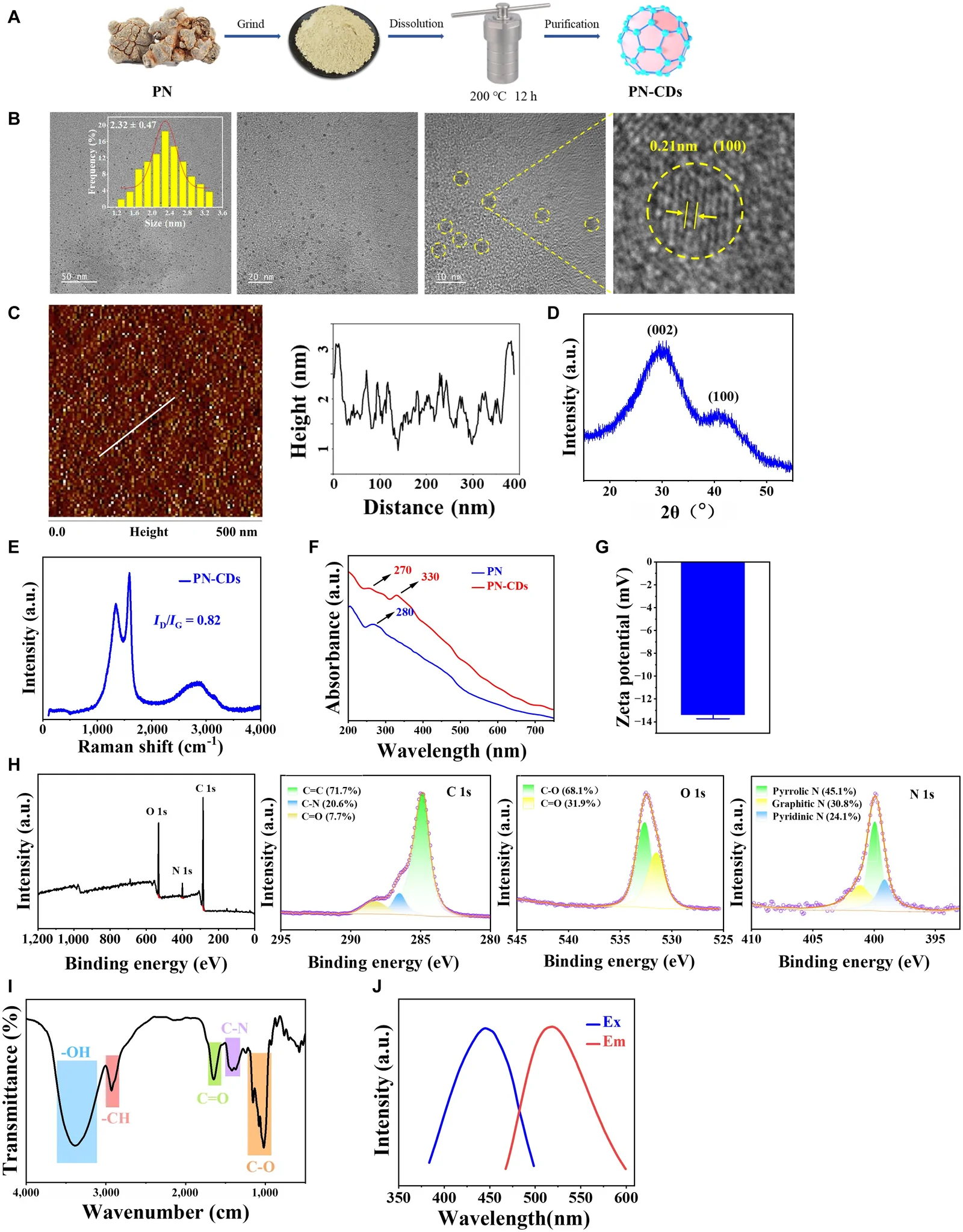
Characterization of PN-CDs. (A) Schematic diagram of PN-CDs synthesis. (B and C) Representative TEM and AFM images of PN-CDs. (D and E) XRD and Raman of PN-CDs. (F) UV–Vis of PN-CDs. (G) The surface charge and stability of PN-CDs were detected by Zeta potential. (H and I) XPS and FTIR were used to analyze the chemical elements and functional groups of PN-CDs. (J) The optical properties of PN-CDs were evaluated using PL.

### Biocompatibility of PN-CDs

Prior to exploring the effect of PN-CDs on CIRI, its biocompatibility was evaluated using CCK-8, Calcein-AM/PI double staining, hemolysis test, BBB permeability assay, blood biochemical analysis, and H&E. As displayed in Fig. 2A, the cell viability of primary neurons and SH-SY5Y was more than 80% with PN-CD concentrations in the range of 0 to 1,000 μg/ml. Consistently, the number of living cells (green) in SH-SY5Y and primary neurons treated with 80, 320, and 640 μg/ml PN-CDs was similar to that in the control group, and only rare dead cells (red) were observed even when the concentration of PN-CDs increased to 640 μg/ml (Fig. 2B). Additionally, the hemolysis rates of different concentrations of PN-CDs (10, 20, 40, 80, 160, 320, 640, and 1,000 μg/ml) were lower than the national standard of 5% (Fig. 2C). To investigate the BBB permeability of PN-CDs, a BBB model was established and used for subsequent experiments on the fifth day (TEER > 200 Ω·cm^2^) (Fig. 2D). Figure 2E indicates that, compared with PN, the BBB permeability of PN-CDs was distinctly increased in a concentration- and time-dependent manner. Usually, ALT, AST, BUN, DBIL, TBIL, UA, ALP, and LDH1 were used to evaluate the function of liver, kidney, and heart. In comparison with the control group, no marked alterations in the levels of these factors were observed in the PN-CDs group (Fig. 2F). Meanwhile, H&E results revealed that PN-CDs did not damage the kidney, lung, liver, heart, and spleen of rats (Fig. 2G).

**Fig. 2.**
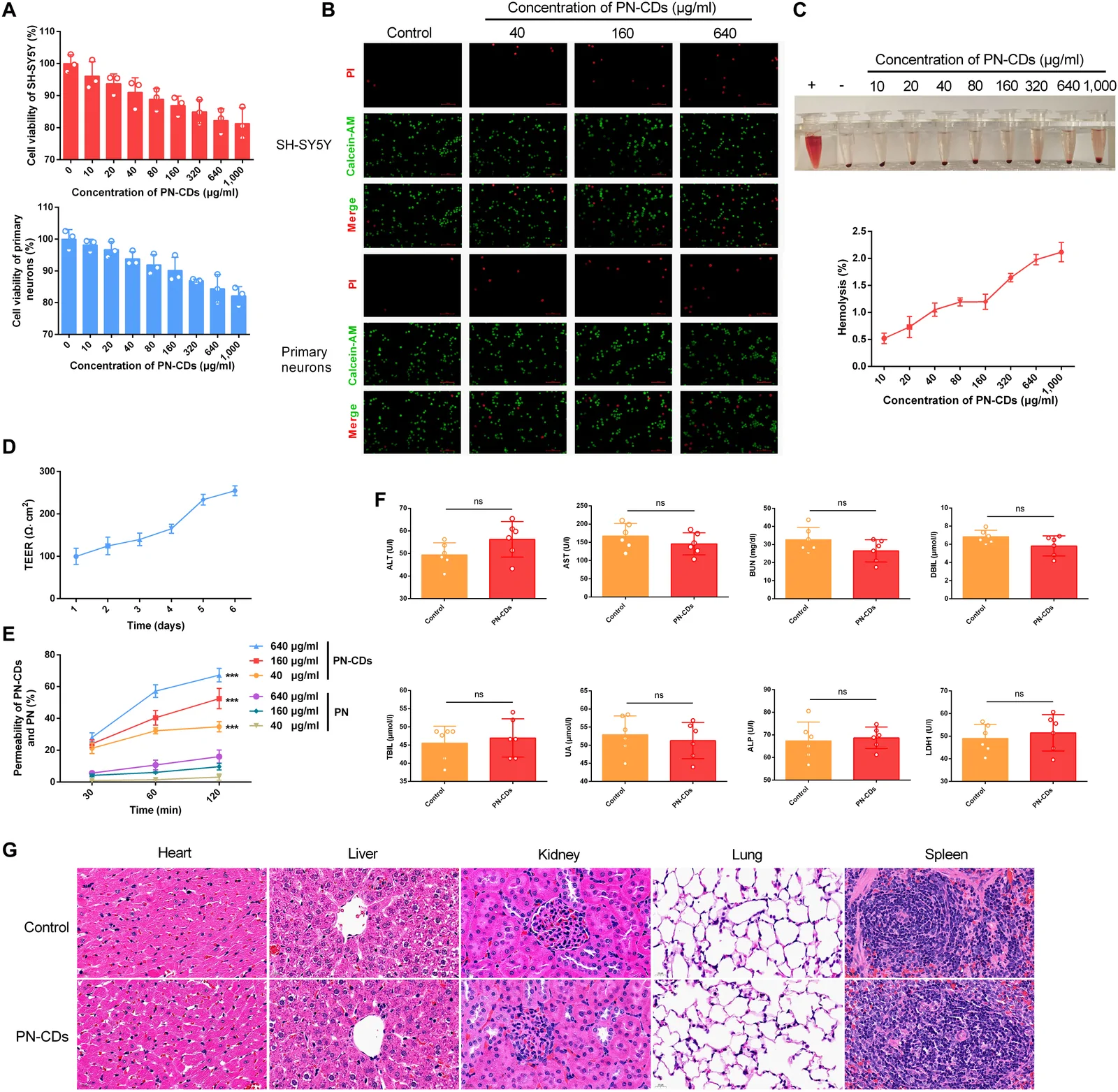
Biocompatibility of PN-CDs. (A) Cell viability of primary neurons and SH-SY5Y was assessed using CCK-8. (B) Calcein-AM/PI double staining images of SH-SY5Y and primary neurons (*n* = 3 independent experiments). (C) Hemolysis test of PN-CDs. (D) TEER of BBB model in vitro. (E) BBB permeability of PN-CDs and PN. (F) The concentrations of AST, ALT, BUN, DBIL, TBIL, UA, ALP, and LDH1 in rat serum. (G) H&E staining of kidney, lung, liver, heart, and spleen in rats. ***P < 0.001; ns, not significant (*n* = 6 for biologically independent animals).

### PN-CDs inhibit OGD/R-induced inflammation of neuronal cells in vitro

In consideration of the negligible toxicity and good biocompatibility of PN-CDs, subsequent functional experiments were performed. As displayed in Fig. 3A, OGD/R treatment markedly elevated the content of IL-1β, IL-6, and TNF-α in primary neurons and SH-SY5Y, while PN-CDs reversed the above changes. qRT-PCR and Western blot analyses also showed that the levels of IL-6, IL-1β, and TNF-α were notably up-regulated in the OGD/R group relative to the control group, but this phenomenon was alleviated by PN-CDs (Fig. 3B and C).

**Fig. 3.**
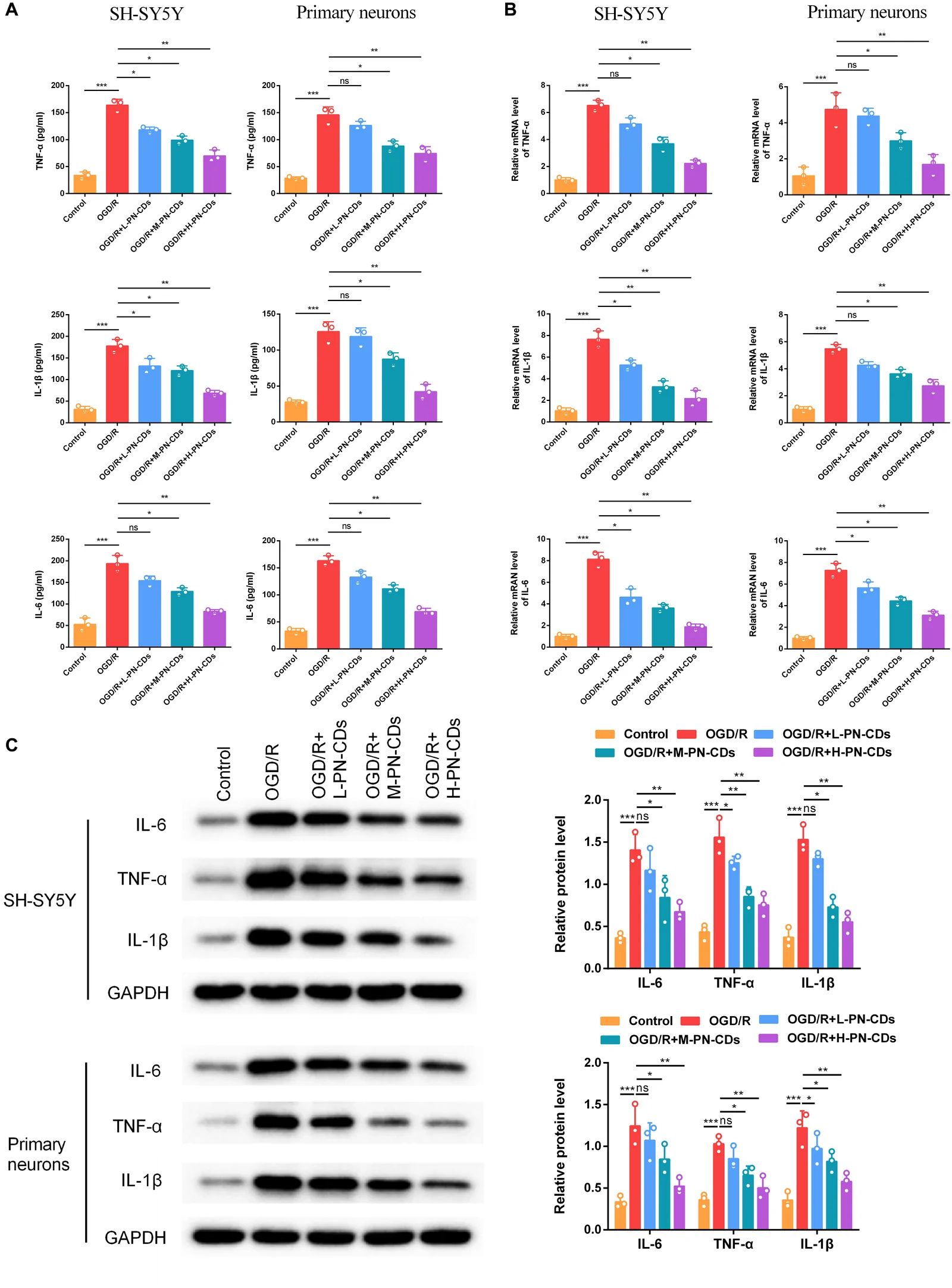
The effect of PN-CDs on OGD/R-induced inflammatory response in neuronal cells. (A) ELISA analysis of inflammatory factors in primary neurons and SH-SY5Y. (B and C) Inflammatory factors were assessed using qRT-PCR and Western blot. *P < 0.05, **P < 0.01, ***P < 0.001; ns, not significant (*n* = 3 independent experiments).

### PN-CDs inhibit OGD/R-induced apoptosis of neuronal cells in vitro

Subsequently, OGD/R-induced SH-SY5Y and primary neurons were incubated with 40, 160, and 640 μg/ml PN-CDs and apoptosis was assessed using flow cytometry and Western blot. As revealed in Fig. 4A, OGD/R treatment obviously promoted the apoptosis of neuronal cells, while PN-CDs alleviated the up-regulation of apoptosis induced by OGD/R. Western blot analysis also indicated that PN-CDs dose-dependently restored the OGD/R-induced reduction in Bcl-2 expression and elevation in Bax and C-caspase-3 levels in neuronal cells (Fig. 4B).

**Fig. 4.**
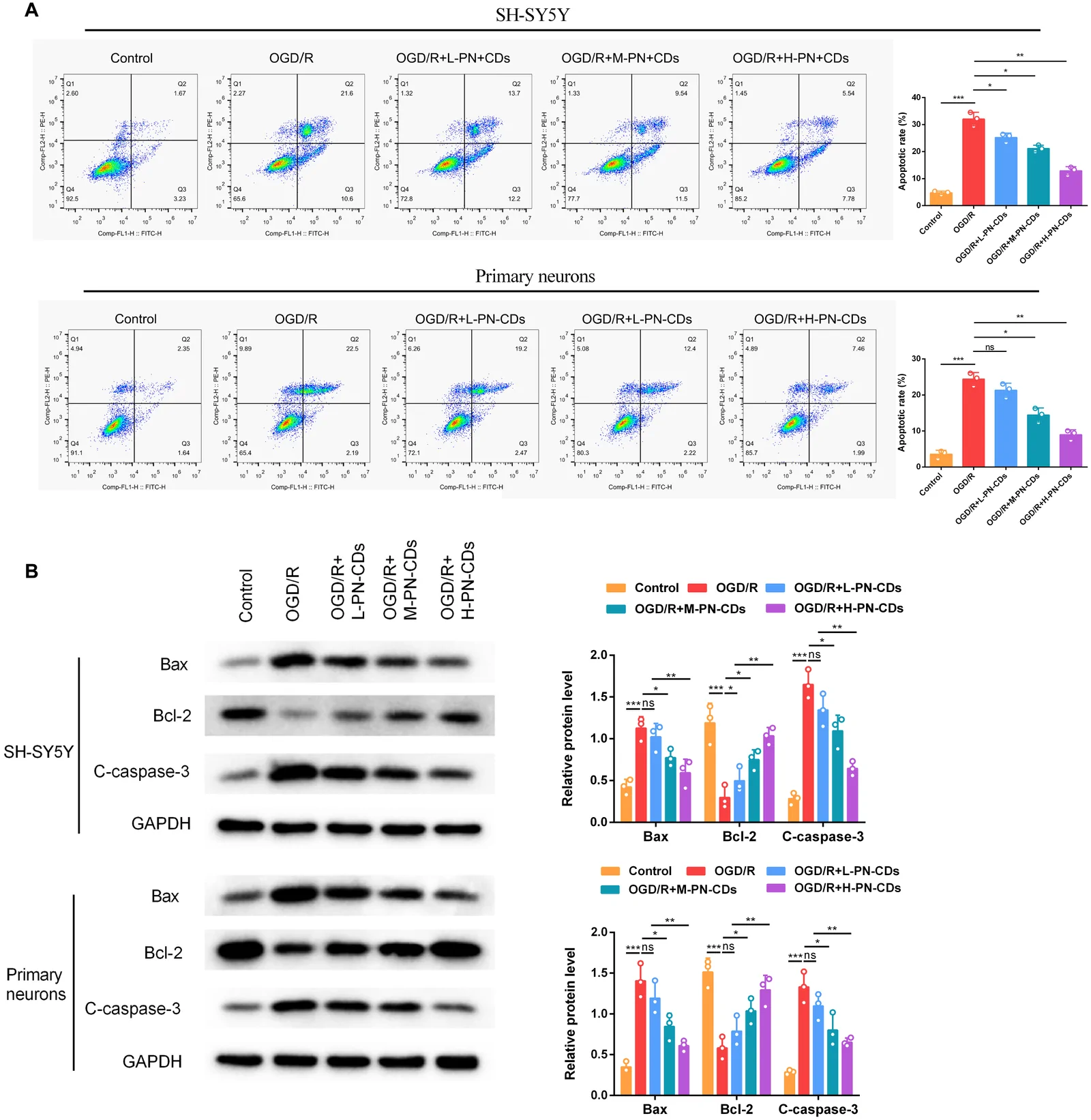
The effect of PN-CDs on OGD/R-induced apoptosis in neuronal cells. (A) The apoptosis of SH-SY5Y and primary neurons was assessed by flow cytometry. (B) Western blot analysis of apoptotic proteins in SH-SY5Y and primary neurons. *P < 0.05, **P < 0.01, ***P < 0.001; ns, not significant (*n* = 3 independent experiments).

### PN-CDs reduce OGD/R-induced oxidative stress of neuronal cells in vitro

Additionally, the impacts of PN-CDs on OGD/R-induced oxidative stress in neuronal cells were evaluated by detecting the levels of ROS, GSH, SOD, and MDA, which were commonly used indicators for assessing the degree of oxidative stress. The ROS level was determined using the DCFH-DA fluorescent probe. As illustrated in Fig. 5A, ROS fluorescence intensity was visibly increased in OGD/R-induced primary neurons and SH-SY5Y, but the above changes were reversed by PN-CDs. Relative to the control group, OGD/R treatment obviously down-regulated GSH and SOD levels and up-regulated MDA level in SH-SY5Y and primary neurons, while PN-CDs inhibited the above phenomena induced by OGD/R (Fig. 5B).

**Fig. 5.**
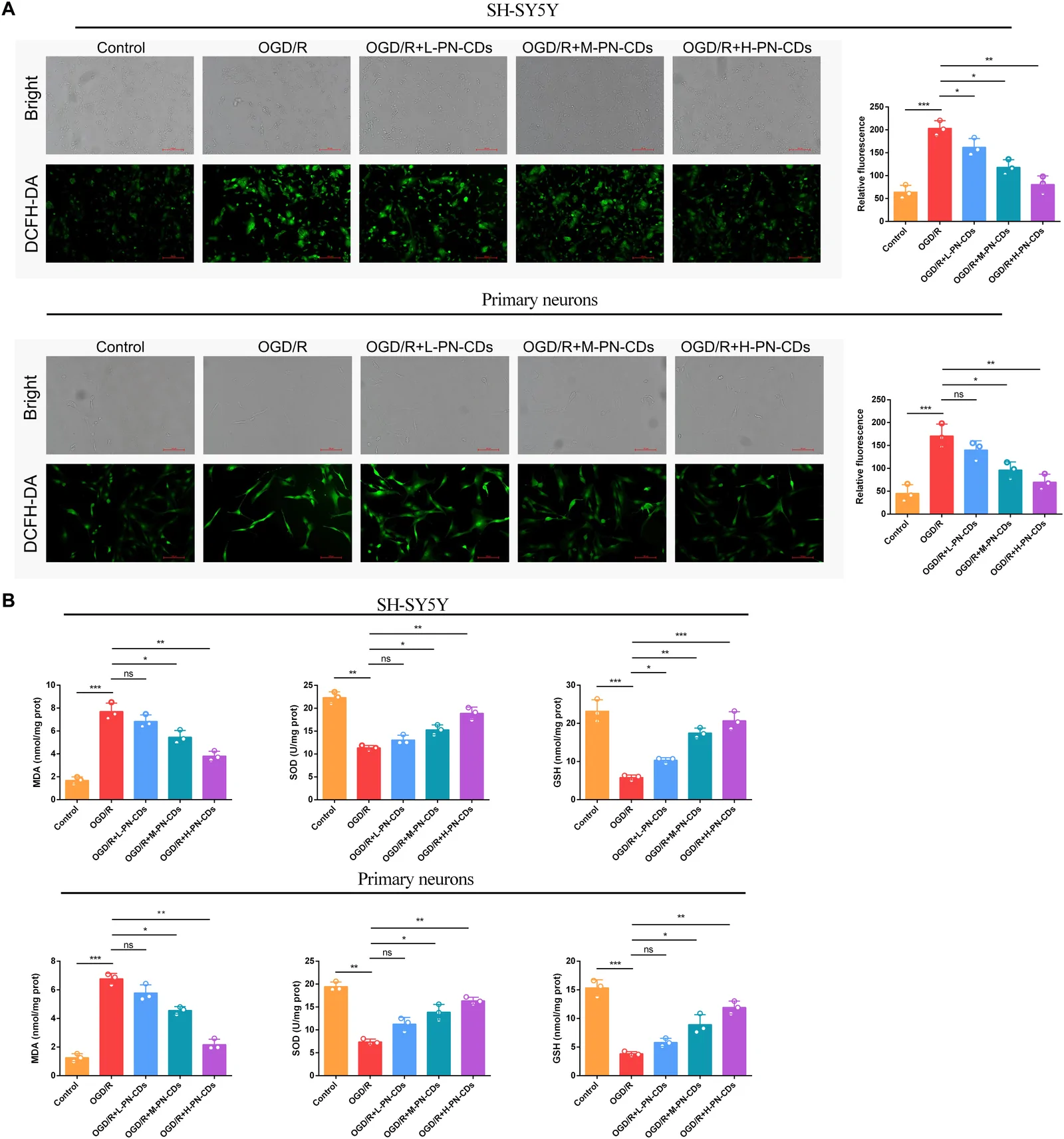
The effect of PN-CDs on OGD/R-induced oxidative stress in neuronal cells. (A) ROS was detected using the DCFH-DA probe. (B) The levels of GSH, SOD, and MDA in neuronal cells. *P < 0.05, **P < 0.01, ***P < 0.001; ns, not significant (*n* = 3 independent experiments).

### PN-CDs alleviate the pathological damage of CIRI rats

SD rats were subjected to MCAO/R surgery, orally administered with PN-CDs (100, 200, and 300 mg/kg/d) for 7 consecutive days, and then neurological function and pathological damage were detected (Fig. 6A). As shown in Fig. 6B, the neurological score of rats in the CIRI group was higher than that in the sham group, but PN-CDs reduced the increase in score caused by CIRI. A higher score represented more serious neurological impairment. TTC results revealed that there was a large area of infarction in the brain tissue of CIRI rats, which was reduced by PN-CDs treatment (Fig. 6C). Besides, defective structure and reduced Nissl were found in the brain tissue of CIRI group rats, but the above damages were improved by PN-CDs (Fig. 6D). TUNEL results also showed that the up-regulation of apoptosis in the rat brain tissues induced by CIRI was reversed by PN-CDs (Fig. 6E).

**Fig. 6.**
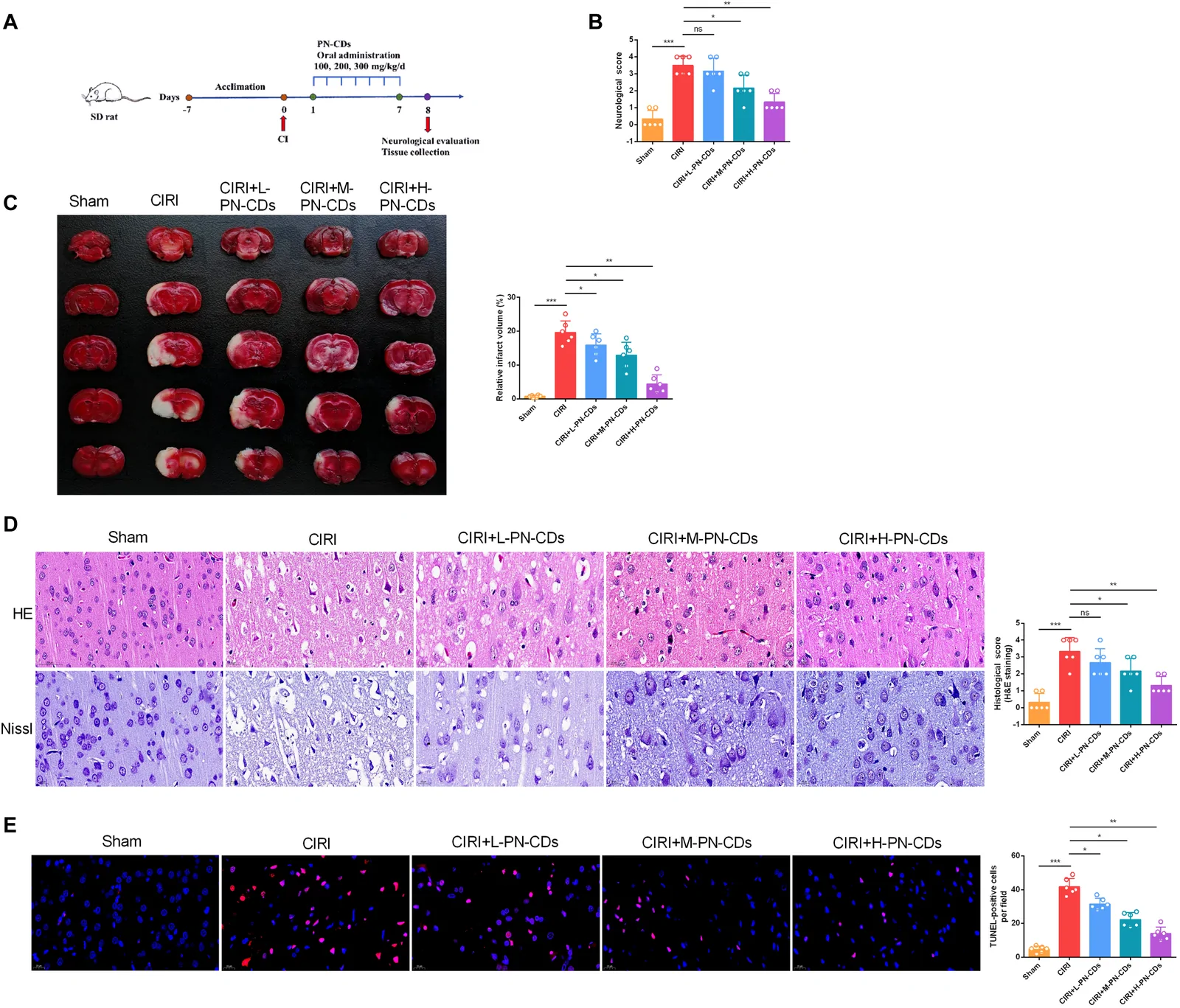
PN-CDs alleviates the pathological damage of CIRI rats. (A) Timeline of rat modeling, drug treatment, and sampling. Specifically, SD rats were purchased, adaptively fed for 7 consecutive days, and then subjected to MCAO/R. After MCAO/R modeling for 24 h, rats were orally administered with PN-CDs (100, 200, or 300 mg/kg/d) for 7 consecutive days. One day after the last administration, rats were evaluated for neurological function, and then brain tissues were collected for further studies. (B) Neurological score of rats. (C) The infarction of rat brain tissues was evaluated using TTC staining. (D) H&E and Nissl staining of rat brain tissues. (E) TUNEL staining of rat brain tissues. *P < 0.05, **P < 0.01, ***P < 0.001; ns, not significant (*n* = 6 for biologically independent animals).

### PN-CDs inhibit inflammation, apoptosis, and oxidative stress in CIRI rats

PN-CDs reduced inflammation, cell death, and oxidative stress caused by OGD/R in neuronal cells in vitro. Accordingly, the levels of apoptosis-related proteins, inflammatory factors, MDA, SOD, and GSH were measured in rats to evaluate the effects of PN-CDs in vivo. As displayed in Fig. 7A–C, the levels of IL-6, IL-1β, and TNF-α were markedly elevated in the CIRI group, and these alterations were effectively mitigated by PN-CDs treatment. Western blot analysis demonstrated that CIRI treatment obviously decreased Bcl-2 level and increased C-caspase-3 and Bax levels, which were reversed by PN-CDs (Fig. 7D). Furthermore, PN-CDs effectively reversed the CIRI-induced decreases in GSH and SOD levels and increases in MDA content in rats. (Fig. 7E).

**Fig. 7.**
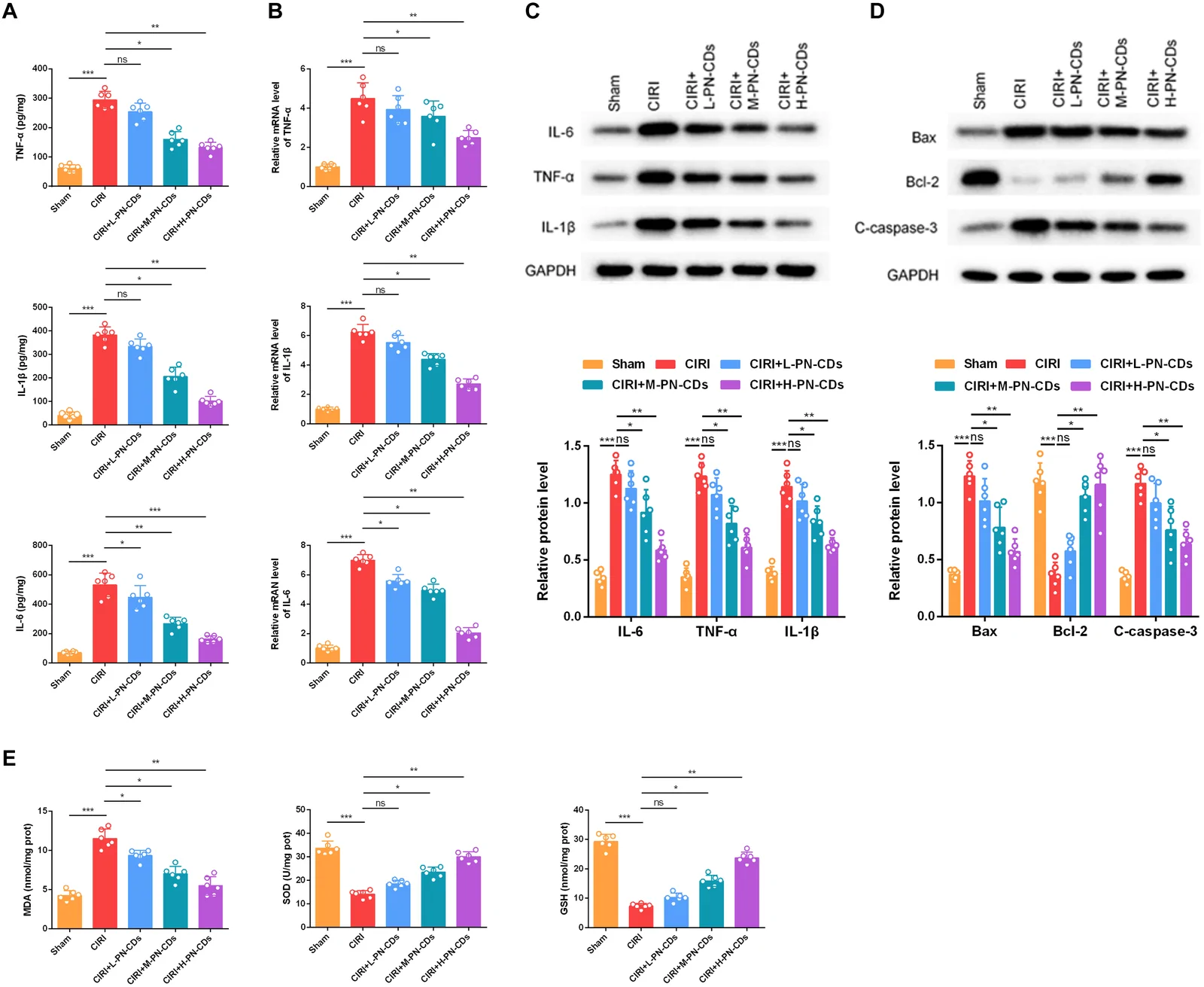
The effect of PN-CDs on inflammation, apoptosis, and oxidative stress in CIRI rats. (A) The concentrations of inflammatory factors in rat brain tissues were measured using ELISA. (B and C) Western blot and qRT-PCR analyses of inflammatory factors in rat brain tissues. (D) Western blot analysis of apoptotic proteins in rat brain tissues. (E) The levels of GSH, SOD, and MDA in rat brain tissues (*n* = 6 for biologically independent animals). *P < 0.05, **P < 0.01, ***P < 0.001; ns, not significant.

### The improvement effect of PN-CDs on CIRI rats was markedly better than that of PN and PNS

The protective effect of PNS on CIRI has been confirmed by numerous scholars [6–8]. Edaravone, a free radical scavenger, is regarded as the first-line drug for ischemic stroke in clinical practice [37–39]. PN-CDs synthesized by PN also markedly improved the damage in CIRI rats. However, it was not clear whether the therapeutic effect of PN-CDs on CIRI rats was better than that of PNS, PN, and edaravone. Hence, CIRI rats were administered with PN-CDs (300 mg/kg/d), PNS (120 mg/kg/d), PN (300 mg/kg/d), or edaravone (6 mg/kg/d) for 7 consecutive days, and neurological function, pathological damage, inflammation, apoptosis, and oxidative stress were evaluated. As illustrated in Fig. 8A, the neurological score of rats in the CIRI + PN-CDs group was lower than that in the CIRI + PNS and CIRI + PN groups, but similar to that in the CIRI + Edaravone group, which indicated that PN-CDs had a better improvement effect on neurological dysfunction in CIRI rats. Compared with the CIRI + PNS and CIRI + PN groups, the infarct volume of brain tissue in CIRI + PN-CDs group rats was visibly reduced. Inversely, there was no marked difference in infarct volume between the CIRI + PN-CDs and CIRI + Edaravone groups (Fig. 8B). H&E staining revealed that a more complete and ordered structure was detected in the brain tissue of CIRI + PN-CDs group rats, which was similar to the CIRI + Edaravone group, indicating that PN-CDs might have a protective effect comparable to that of edaravone (Fig. 8C). Moreover, the CIRI + PN-CDs group displayed an increased number of Nissl bodies and a reduced number of TUNEL-positive cells in rat brain tissue relative to the CIRI + PNS and CIRI + PN groups, and comparable to the CIRI + Edaravone group (Fig. 8C). Compared with PNS and PN, PN-CDs treatment obviously down-regulated IL-6, IL-1β, TNF-α, Bax, C-caspase-3, and MDA levels and up-regulated Bcl-2, SOD, and GSH levels in CIRI rats. Meanwhile, PN-CDs exhibited anti-inflammatory and antioxidative stress effects similar to those of edaravone (Fig. 8D and E). Altogether, the therapeutic effect of PN-CDs on CIRI rats was obviously better than that of PNS and PN, and comparable to that of the first-line drug edaravone.

**Fig. 8.**
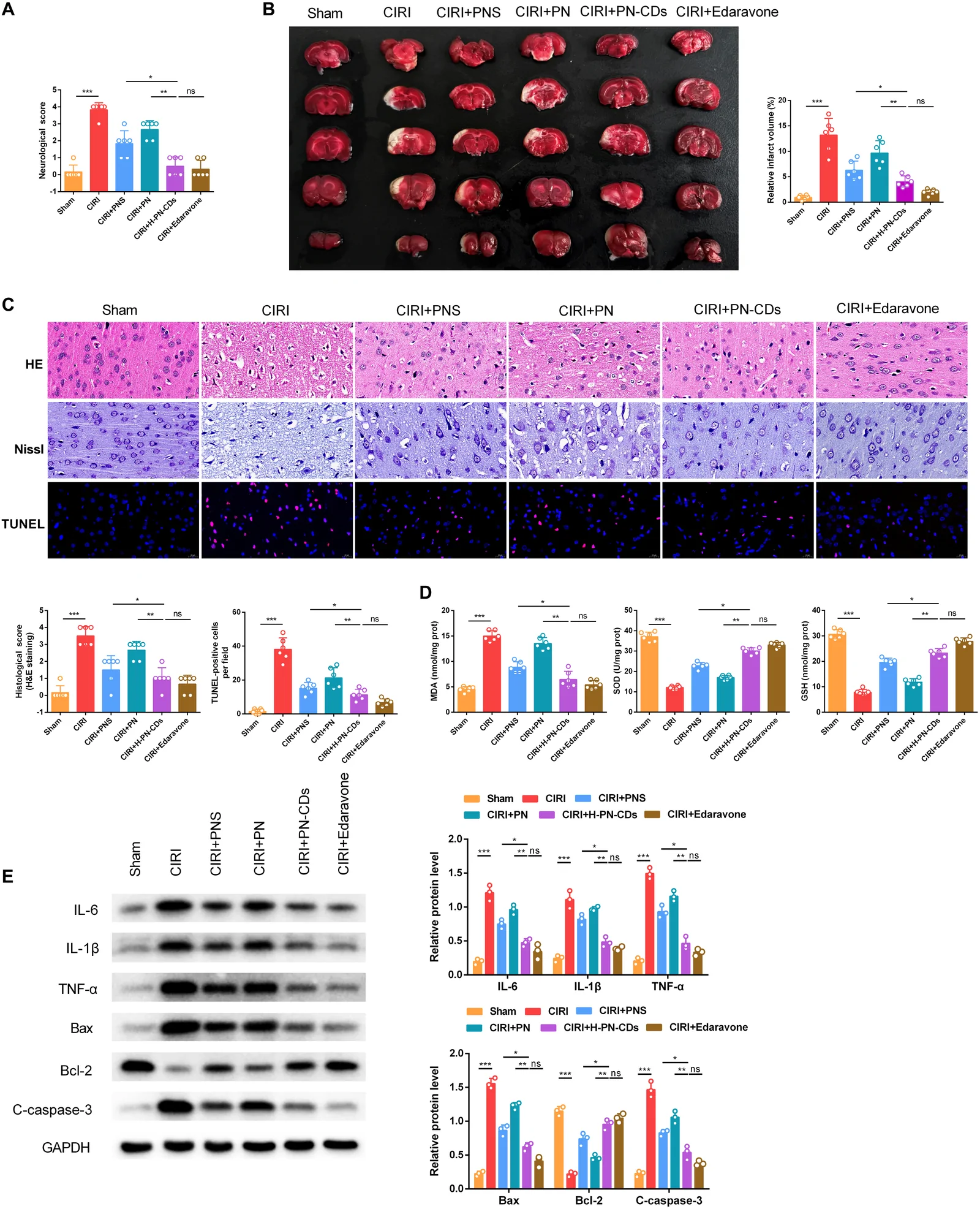
The therapeutic effect of PN-CDs, PN, PNS, and edaravone on CIRI rats. CIRI rats were administered with PN-CDs, PNS, PN, or edaravone for 7 consecutive days, and neurological function, pathological damage, inflammation, apoptosis, and oxidative stress were evaluated. (A) Neurological score of rats. (B) Rat brain tissues stained with TTC. (C) H&E, Nissl, and TUNEL staining of rat brain tissues. (D) The levels of GSH, SOD, and MDA in rat brain tissues. (E) Western blot analysis of inflammatory and apoptotic proteins in rat brain tissues. *P < 0.05, **P < 0.01, ***P < 0.001; ns, not significant (*n* = 6 for biologically independent animals).

## Discussion

The interruption of blood supply to the brain induces ischemia and hypoxic necrosis of local brain tissues and ultimately impairs neurological function, which is defined as ischemic stroke. Restoring blood flow represents the primary clinical approach for managing ischemic stroke, but it usually causes CIRI. Actively exploring drugs for the treatment of CIRI has become the focus of research. PN possesses notable pharmacological properties, including blood circulation activation, blood stasis dissipation, and anti-inflammatory effects. Besides, PNS, the active ingredient of PN, has been verified to alleviate CIRI. Recently, CQDs have exhibited distinct therapeutic benefits in the management of CIRI. The purpose of this project was to synthesize PN-CDs and clarify their role in CIRI. Our results indicated that PN-CDs were successfully synthesized and inhibited inflammation, apoptosis, oxidative stress, and pathological damage in CIRI.

At present, the therapeutic efficacy of neuroprotective drugs for CIRI is largely limited by inadequate BBB permeability, low bioavailability, and systemic adverse effects, all of which pose critical obstacles to their clinical application. As a novel nanocarrier, CQDs offer a promising strategy to circumvent the above-mentioned limitations. Thus, PN-CDs were synthesized and their BBB permeability was further evaluated. The results showed that the BBB permeability of PN-CDs was distinctly increased in a concentration- and time-dependent manner as compared with PN, but the underlying mechanism remains to be elucidated. PN-CDs, owing to their relatively small particle size, may enter the brain parenchyma via paracellular passive diffusion or transcellular vesicular trafficking pathways. Moreover, the abundant functional groups on the surface of PN-CDs may facilitate their uptake by brain microvascular endothelial cells via clathrin- or caveolin-mediated endocytosis, thereby enabling transcytosis across the BBB. Collectively, we speculate that PN-CDs may regulate BBB transport through a dual mechanism. The relative contribution of each mechanism and their interplay warrant further investigation. In future studies, we will further elucidate the molecular transport mechanism of PN-CDs across BBB by pathway inhibition, colocalization analysis, and fluorescence tracing.

Inflammatory response is a crucial pathological mechanism of CIRI. After CIRI, inflammatory cells are activated by oxygen free radicals and other messengers, and then release adhesion and chemokines molecules, destroy endothelial cells and BBBs, and then cause brain edema and neuronal damage. Targeting inflammatory cascades has been increasingly recognized as an effective clinical option for CIRI. Li et al. [40] constructed CIRI rat and cell models and treated them with nuciferine and found that it inhibited the release of inflammatory factor, thereby improving cognitive and motor dysfunction and reducing infarct volume. Baicalein extracted from *Scutellaria baicalensis* Georgi has marked anti-inflammatory effects. Xie et al. [41] pointed out that baicalein alleviated neuroinflammation in CIRI. Herein, CIRI increased the levels of TNF-α, IL-6, and IL-1β in neuronal cells and rats, while PN-CDs reversed the above changes. In short, CIRI-elicited inflammatory response was markedly inhibited by PN-CDs.

Apoptosis refers to the active and orderly cell death produced by gene regulation under physiological or pathological conditions to preserve homeostatic balance. Recently, the key role of apoptosis in CIRI has attracted the attention of scholars. Xu et al. [42] revealed that Orexin-A alleviated neurological impairment in CIRI rats by inhibiting apoptosis. Saftoside is a monoterpene aldehyde compound extracted from *Crocus sativus*. In CIRI rat and cell models, saftoside promoted autophagy, reduced apoptosis, and alleviated pathological damage by modulating the AMPK/mTOR pathway [43]. In addition, daucosterol palmitate inhibited apoptosis and improved neurological dysfunction, which showed a good application potential in the treatment of CIRI [44]. Herein, OGD/R treatment obviously promoted the apoptosis of neuronal cells, while PN-CDs inhibited the increase of apoptosis. Meanwhile, up-regulated C-caspase-3 and Bax and down-regulated Bcl-2 were observed in OGD/R-induced primary neurons and SH-SY5Y and CIRI rats, but this phenomenon was reversed by PN-CDs. In brief, PN-CDs inhibited apoptosis in CIRI.

Ischemic stroke leads to mitochondrial dysfunction, reduces adenosine triphosphate production, and releases a large amount of ROS. After reperfusion, blood flow recovery causes protein oxidation, DNA damage, and lipid peroxidation, which aggravates neuronal damage. Targeted inhibition of oxidative stress offers a novel strategy for the intervention of CIRI. Lan et al. [45] demonstrated that isoliquiritigenin attenuated oxidative stress, improved mitochondrial dysfunction, alleviated neurological deficits, and decreased cerebral infarction volume as well as neuronal apoptosis through activating the Nrf2 pathway. Amifostine improved neurological function and cerebral infarction in CIRI rats via p38-mediated mitochondrial dysfunction and oxidative stress [46]. In this project, CIRI obviously up-regulated ROS and MDA, and decreased GSH and SOD in neuronal cells and rats, while PN-CDs reversed the above phenomena in a concentration-dependent manner. Notably, the differences in electron-donating capacity among various N configurations may be associated with ROS-scavenging performance. Pyrrolic N and pyridinic N act as electron-rich sites and regulate ROS scavenging by donating electrons or facilitating electron transfer. Graphitic N enhances the electrical conductivity of the carbon skeleton, thereby accelerating electron transfer. High-resolution N1s XPS spectra with peak fitting showed that pyridinic N, pyrrolic N, and graphitic N account for 24.1%, 45.1%, and 30.8%, respectively. It is hypothesized that the antioxidant capacity of PN-CDs arises from the high proportion of pyrrolic N/pyridinic N and its synergistic interplay with graphitic N. In a word, PN-CDs inhibited oxidative stress in CIRI.

Notably, inflammation, apoptosis, and oxidative stress are not isolated in CIRI, but interact to form a complex crosstalk network. CIRI induces excessive accumulation of ROS, activates inflammatory and mitochondria-related pathways, and ultimately triggers an inflammatory response and initiates apoptosis. Furthermore, the continuously activated inflammatory response further promotes oxidative stress and directly induces apoptosis. The apoptotic cells release DAMPs and exacerbate inflammation and oxidative stress to form a positive feedback loop. In this study, PN-CDs inhibited oxidative stress, inflammation, and apoptosis after CIRI. Based on the cascade amplification relationship among oxidative stress, inflammation, and apoptosis, we hypothesize that the protective effect of PN-CDs on CIRI might be achieved by blocking oxidative stress, thereby inhibiting inflammation and apoptosis. Interestingly, Gao et al. [9] demonstrated that the catalytic activity of CQDs arises from the sizable sp^2^ plane and oxygen-bearing surface groups, and CQDs can effectively eliminate ROS, thereby exerting anti-apoptotic and anti-inflammatory effects. Therefore, O–H and C=O might be involved in the antiapoptotic and anti-inflammatory effects of PN-CDs. Besides, surface passivation might affect bioactivity by altering the density and accessibility of these active oxygen-containing groups; excessive passivation might reduce electron transfer capacity and diminish ROS scavenging efficacy, while mild or targeted passivation could potentially enhance stability or specificity.

PNS is one of the effective components of PN and possesses hemostatic, antiaging, anti-inflammatory, and antitumor effects. Numerous investigations have substantiated the pivotal involvement of PNS in the therapeutic management of CIRI. Dong et al. [47] found that PNS treatment improved neurological function, energy metabolism, and motor coordination and reduced infarct volume in CIRI rats. Furthermore, Wu et al. [8] revealed that PNS markedly reduced infarct volume and histopathological changes, inhibited oxidative stress, and enhanced neurological function. Edaravone, first launched in Japan in 2001, is a neuroprotective agent, which inhibits lipid peroxidation, inflammatory responses, and cell apoptosis by eliminating oxygen free radicals, thereby exerting a protective effect on nerve cell damage caused by cerebral ischemia [37]. Notably, edaravone has become a classic drug for treating ischemic stroke. Herein, CIRI rats were administered with PN-CDs, PNS, PN, or edaravone to investigate whether PN-CDs had the best effect. Compared with PNS and PN, PN-CDs treatment obviously reduced infarct volume, improved neurological dysfunction, alleviated histopathological damage, and inhibited inflammation, apoptosis, and oxidative stress in CIRI rats. Interestingly, PN-CDs exhibited a similar neuroprotective effect against CIRI to that of edaravone. In short, the therapeutic effect of PN-CDs on CIRI rats was obviously better than that of PNS and PN, and comparable to that of the first-line drug edaravone, indicating that it might be a new and effective drug for treating CIRI.

Altogether, we successfully synthesized novel and effective PN-CDs and found that they alleviated CIRI by inhibiting inflammation, apoptosis, and oxidative stress. These results offer a new approach for treating CIRI. Although this study revealed that PN-CDs improved CIRI, the molecular mechanism was unknown. Specifically, PN-CDs function by scavenging ROS or activating endogenous antioxidant enzymes. Therefore, we would perform ROS measurement, antioxidant enzyme activity evaluation, and pathway-specific inhibitors to elucidate the underlying mechanism of PN-CDs in CIRI protection. Besides, butylphthalide and rtPA are also regarded as the first-line drugs for CIRI. Our study only compared the therapeutic effects of edaravone and PN-CDs, but the comparison with butylphthalide and rtPA remains to be further investigated. In clinical applications, PN often requires prolonged administration (weeks or even months). To more comprehensively assess the long-term safety and efficacy persistence of PN-CDs, we would carry out a longer period of drug administration experiments in subsequent studies.

This study systematically investigates the protective effects of PN-CDs on CIRI. As a novel and efficient administration strategy, PN-CDs alleviate CIRI by inhibiting inflammation, apoptosis, and oxidative stress. Our findings demonstrate that PN-CDs possess great potential as an effective neuroprotective agent for CIRI treatment, providing new insights for the development of natural CQD-based therapies for ischemic stroke.

## Ethical Approval

The Ethics Committee of the Affiliated Hospital of Shandong University of Traditional Chinese Medicine approved the study (20240108001). All animal experiments were performed in accordance with the ARRIVE guidelines.

## Data Availability

The datasets used and analyzed during the current study are available from the corresponding authors on reasonable request.
